# Clinical outcomes and biomechanics in bicruciate‐retaining total knee arthroplasty

**DOI:** 10.1002/jeo2.70152

**Published:** 2025-01-26

**Authors:** Kenichi Kono, Tomofumi Kage, Takaharu Yamazaki, Shuji Taketomi, Ryota Yamagami, Hiroshi Inui, Tetsuya Tomita, Sakae Tanaka

**Affiliations:** ^1^ Department of Orthopaedic Surgery, Faculty of Medicine The University of Tokyo Tokyo Japan; ^2^ Department of Information Systems Faculty of Engineering, Saitama Institute of Technology Saitama Japan; ^3^ Department of Orthopaedics, Saitama Medical Center Saitama Medical University Saitama Japan; ^4^ Department of Orthopaedic Biomaterial Science Osaka University Graduate School of Medicine Osaka Japan; ^5^ Master Course of Health Sciences, Graduate School of Health Sciences Morinomiya University of Medical Sciences Osaka Japan

**Keywords:** bicruciate‐retaining total knee arthroplasty, kinematics, patient‐reported outcome measures, totation

## Abstract

**Purpose:**

To clarify the influence of biomechanics on post‐operative clinical outcomes in bicruciate‐retaining total knee arthroplasty (BCR‐TKA).

**Methods:**

Severe medial osteoarthritis who underwent BCR‐TKA were examined. Each patient was asked to perform a squat (weight‐bearing [WB]) and active assisted knee flexion (non‐WB [NWB]) under single fluoroscopy surveillance. A 2D‐to‐3D registration technique was used. Patients were divided into two groups based on their 1‐year post‐operative patient‐reported outcome measures (PROMs) using hierarchical cluster analysis. The rotational alignment on computed tomography, anterior stability at 30° of knee flexion, axial rotation of the femur relative to the tibial component and anteroposterior translation of the medial and lateral femorotibial contact points were measured.

**Results:**

Components did not significantly differ between the groups, with 1.6 ± 5.0° and 5.4 ± 4.7° of femoral internal rotation in the low PROM (*N* = 28) and high PROM (*N* = 8) groups, respectively. Moreover, anterior stability did not significantly differ (low PROM: 4.9 ± 1.4 mm, high PROM: 5.3 ± 1.0 mm). The knee externally rotated from 0° to 70° and from 50° to 110° of flexion during WB and NWB, respectively. The low‐PROM group exhibited more external rotation across all ranges of motion. Medial contact points moved backwards from 0° to 30° of flexion during WB, forward from 30° to 100° of flexion, and backwards from 100° to 110° of flexion. The low‐PROM group was positioned more forward throughout the full range of motion during WB. Lateral contact points moved backwards at 0−30° of flexion, forward at 70−100° of flexion, and backwards at 100−110° of flexion during WB, while there was backward movement at 50° of flexion during NWB. Both activities exhibited a more posterior position in the low‐PROM group throughout the full range of motion.

**Conclusion:**

The femoral component in the low‐PROM group was externally rotated across all ranges of motion, and the lateral contact points were posteriorly located in BCR‐TKA.

**Level of Evidence:**

Level II, prospective cohort study.

**Abbreviations**
ACL: anterior cruciate ligament
AP: anteroposterior
BCR‐TKA: bicruciate‐retaining TKA
BCS‐TKA: bicruciate‐stabilised TKA
EZR: Easy R
FC: femoral component
GR: Group
KOOS: Knee Injury and Osteoarthritis Outcome Score
KSS: Knee Society Score
NWB: non‐weight‐bearing
OA: osteoarthritis
PCL: posterior cruciate ligament
PROM: patient‐reported outcome measure
TC: tibial component
TKA: total knee arthroplasty
WB: weight‐bearing

## INTRODUCTION

Recently, the average rate of patient dissatisfaction following total knee arthroplasty (TKA) has improved by approximately 10% [2]. The most common sociodemographic factors for dissatisfaction are age <65 years, lower income, and non‐white ethnicity [2]. Preoperative factors include lower Kellgren−Lawrence scores, depression/anxiety and pain catastrophising [2]. However, an increased number of younger patients with osteoarthritis (OA) have been undergoing TKA, suggesting that these younger patients with OA, including those with an intact anterior cruciate ligament (ACL), are inclined to be dissatisfied after surgery.

Although approximately 55% on average of patients have an intact ACL at the time of TKA [21], resection of the remaining ACL during TKA is associated with decreased patient satisfaction [6]. In contrast, bicruciate‐retaining TKA (BCR‐TKA) provides better anteroposterior (AP) stability [12]. For example, Chevalier et al. reported that the native kinematics of the BCR‐TKA knees were comparable to the bicruciate‐stabilised TKA (BCS‐TKA) knees for internal/external rotation and AP translation during staircase descent [1]. In addition, several studies have reported that BCR‐TKA knee kinematics are similar to normal knee kinematics [11, 23]. However, BCR‐TKA knee kinematics do not entirely resemble normal and preoperative knee kinematics [9, 10].

Patient‐reported outcome measures (PROMs) are related to post‐operative kinematics in TKA [13, 25]. Van Onsem et al. reported that during closed kinetic chain movements, patients with poor PROM scores after TKA experience more anterior translation on the medial side and less posterior translation on the lateral side in deep flexion [25]. Moreover, in BCS‐TKA, the higher PROM group was reported to have a smaller external rotation of the femur [13]. However, it remains unclear whether PROMs are related to BCR‐TKA knees. Therefore, the present study aimed to clarify the influence of biomechanics on post‐operative clinical outcomes in BCR‐TKA. We hypothesised that post‐operative biomechanics influence clinical outcomes, such as PROMs, in BCR‐TKA.

## METHODS

A total of 36 knees who underwent BCR‐TKA were examined. The inclusion criteria for this study were as follows: (1) varus knee OA (hip‐knee‐ankle [HKA] angle <180°) and preoperative varus deformity <15°; (2) intact cruciate and collateral ligament; (3) preoperative flexion contracture <15°; (4) primary TKA using the Journey II XR implant; and (5) consent for fluoroscopic evaluation. The exclusion criteria were as follows: (1) valgus knee OA (HKA angle >180°); (2) posttraumatic OA; (3) inflammatory arthritis (e.g., rheumatoid arthritis); (4) revision TKA; and (5) no consent for fluoroscopic evaluation. Patients who could safely perform a deep‐standing squat after surgery were examined. Each patient was asked to perform a deep‐standing squat (weight‐bearing [WB]) and active assisted knee flexion (non‐weight‐bearing [NWB]) at a natural pace under single fluoroscopy surveillance in the sagittal plane. Both activities were performed from full extension to maximum flexion. The participants practised the motion several times before it was recorded. The motions were recorded as sequential digital radiographic images (1024 × 1024 × 12 bits/pixel, 7.5‐Hz serial spot images in a DICOM file) using a 17‐in. flat panel detector system. All images were processed using dynamic range compression for edge enhancement. To estimate the spatial position and orientation of the femoral and tibial components, a 2D‐to‐3D registration technique was used [26]. This technique is based on a contour‐based registration algorithm that uses single‐view fluoroscopic images and 3D computer‐aided design models. The margin of error of the estimated relative motion between the metal components was ≤0.5° for rotation and ≤0.4° for translation [26]. The following variables were measured: knee flexion angle, axial rotation of the femoral component (FC) relative to the tibial component (TC) and AP translation of the medial and lateral femorotibial contact points. Flexion and external rotation of the FC relative to the TC were denoted as positive values.

Positive or negative values of AP translation were defined as anterior or posterior to the axes of the TC, respectively. All data are expressed as the means ± standard deviation. The patients were divided into two groups based on the 1‐year post‐operative PROMs, including the Knee Injury and Osteoarthritis Outcome Score (KOOS) [18] and the 2011 Knee Society Score (KSS 2011) [22]. Based on a hierarchical cluster analysis [13], Group 1 (GR1) comprised patients with low‐to‐medium PROM scores (N = 28), and Group 2 (GR2) comprised patients with good‐to‐excellent PROM scores (N = 8). Additionally, the following parameters were compared between the two groups: rotational alignment on computed tomography (FC angle relative to the surgical epicondylar axis, TC angle relative to the Akagi's line, and FC angle relative to the TC) at 2 weeks post‐operatively [7], anterior stability at 30° of flexion using a KT2000 arthrometer (MEDmetric) with a 134‐N anterior force applied to the proximal tibia [4], and kinematics (knee flexion, rotation angles and AP translation). Fluoroscopic analysis was performed at 10.8 ± 4.6 months after surgery.

### Statistical analyses

Commercial software (SPSS version 25; IBM Corp.) was used to analyse the data. All data are expressed as means ± standard deviations. Two‐way analysis of variance and post hoc pairwise comparisons (Bonferroni test) were used to compare the rotation and varus–valgus angles between GR1 and GR2. The Mann−Whitney test was used to compare the PROMs, patient characteristics, rotational alignment on CT, and anterior stability between the two clusters. *p* Values < 0.05 were used to denote statistical significance. A priori power analysis was performed using G*Power (version 3.1.9.7; Heinrich Heine University) [3] before this study. Seven knees were required to achieve an alpha, power and effect size of 0.05, 0.8 and 0.25, respectively.

## RESULTS

The PROMs and patient characteristics are presented in Tables 1 and 2. The FC was placed 1.1 ± 2.3° externally in GR1 and 0.1 ± 3.0° internally in GR2, with no significant difference between the groups. The TC was placed 3.5 ± 4.8° externally in GR1 and 6.7 ± 5.6° externally in GR2, with no significant difference between them. There was no significant difference in components between the two groups, with 1.6 ± 5.0° of internal rotation in GR1 and 5.4 ± 4.7° in GR2 (Table 3). There was also no significant difference in anterior stability (GR1: 4.9 ± 1.4 mm, GR2: 5.3 ± 1.0 mm) (Table 3).

**Table 1:** Patient‐reported outcome measures (PROMs).

PROMs	GR1	GR2	*p*
KOOS			
Pain	84.7 ± 10.7	97.2 ± 2.4	<0.01
Symptoms	84.2 ± 8.6	95.5 ± 3.0	<0.01
ADL	86.3 ± 10.0	99.1 ± 1.3	<0.01
Sports/recreations	56.6 ± 20.6	91.9 ± 8.6	<0.01
QOL	68.8 ± 16.8	98.4 ± 2.7	<0.01
KSS2011			
Symptoms	19.9 ± 3.0	24.5 ± 1.0	<0.01
Satisfaction	28.9 ± 6.5	39.3 ± 1.4	<0.01
Expectations	9.5 ± 1.8	13.8 ± 1.6	<0.01
Functional activities	73.4 ± 15.3	96.3 ± 2.8	<0.01

Abbreviations: ADL, activity of daily living; GR1, patients with low‐to‐medium PROM scores; GR2, patients with good‐to‐excellent PROM scores; KOOS, Knee Injury and Osteoarthritis Outcome Score; KSS2011, 2011 Knee Society Score; QOL, quality of life.

**Table 2:** Patients' characteristics.

Variables	GR1	GR2	*p*
Age (years)	73.7 ± 6.7	74.1 ± 4.0	0.94
BMI (kg/m^2^)	25.5 ± 2.9	23.9 ± 3.0	0.34
Post‐operative fluoroscopic analysis (months)	11.2 ± 5.0	9.5 ± 2.1	0.47
Sex (males:females)	7:21	2:6	0.88
Coronal alignment (mechanical:functional)	14:14	4:4	1.00
Preoperative hip–knee–ankle angle (°)	172.8 ± 4.6	173.1 ± 6.6	0.73
Post‐operative hip–knee–ankle angle (°)	178.1 ± 2.6	176.3 ± 1.6	0.05
Extension angle (°)	7.5 ± 5.6	10.7 ± 4.0	0.16
Flexion angle (°)	109.7 ± 15.2	119.5 ± 7.5	0.07

Abbreviations: BMI, body mass index; GR1, patients with low‐to‐medium PROM scores; GR2, patients with good‐to‐excellent PROM scores; PROM, patient‐reported outcome measure.

**Table 3:** Rotational alignment on computed tomography and anterior stability.

Variables	GR1	GR2	*p*
FC rotation (°)	1.1 ± 2.3	−0.1 ± 3.0	0.34
TC rotation (°)	3.5 ± 4.8	6.7 ± 5.6	0.18
FC rotation relative to TC (°)	−1.6 ± 5.0	−5.4 ± 4.7	0.06
Anterior stability (mm)	4.9 ± 1.4	5.3 ± 1.0	0.42

Abbreviations: FC, femoral component; GR1, patients with low‐to‐medium PROM scores; GR2, patients with good‐to‐excellent PROM scores; TC, tibial component.

**Figure 1:**
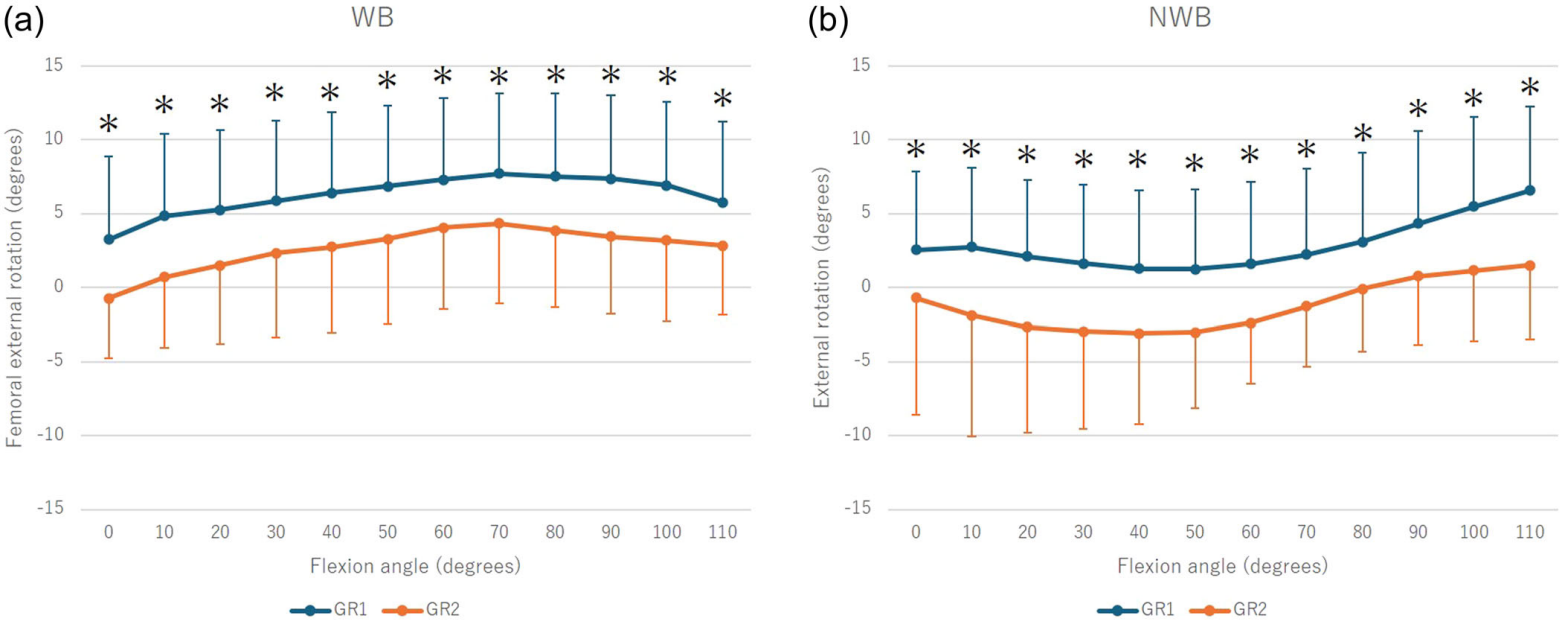
Kinematic comparison between Groups 1 and 2 in terms of the rotation angle during WB (a) and NWB (b). *Significant difference between Groups 1 and 2 (*p* < 0.05). NWB, non‐weight‐bearing; WB, weight‐bearing.

**Figure 2:**
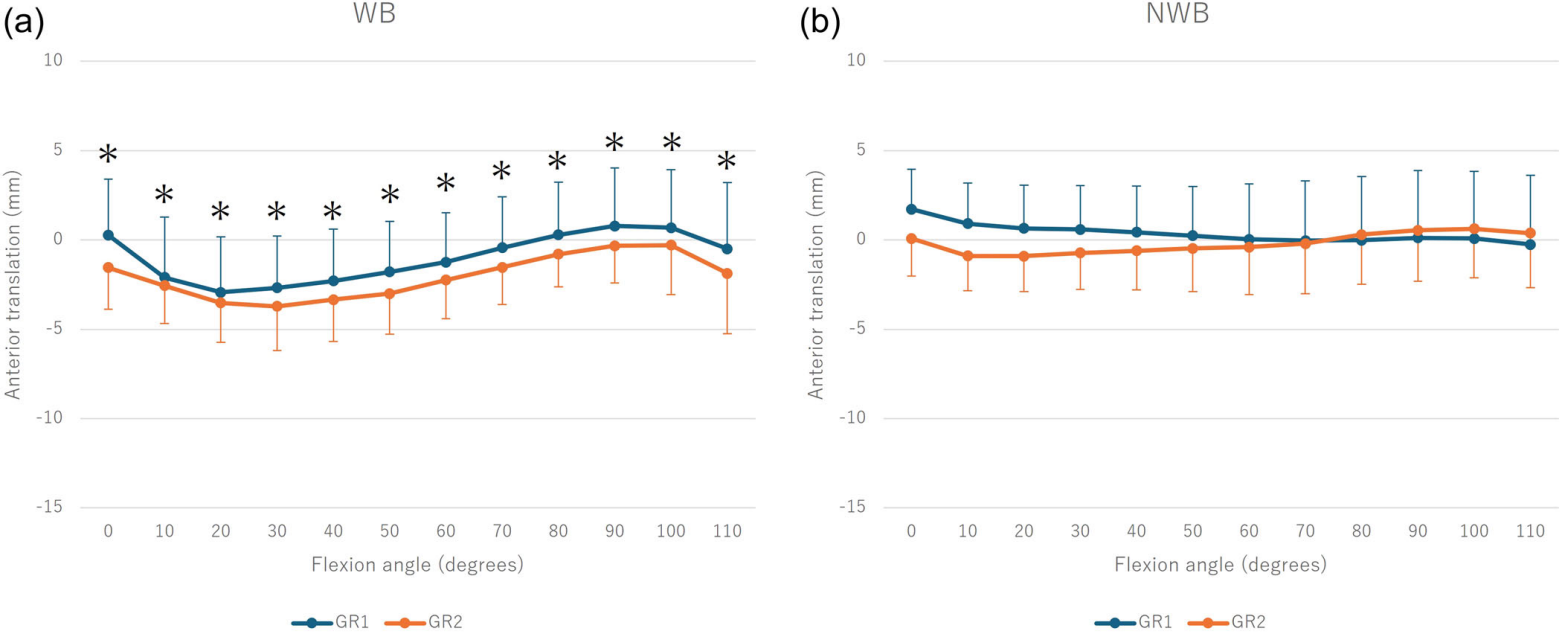
Kinematic comparison between Groups 1 and 2 regarding medial anteroposterior translation during WB (a) and NWB (b). *Significant difference between Groups 1 and 2 (*p* < 0.05). NWB, non‐weight‐bearing; WB, weight‐bearing.

**Figure 3:**
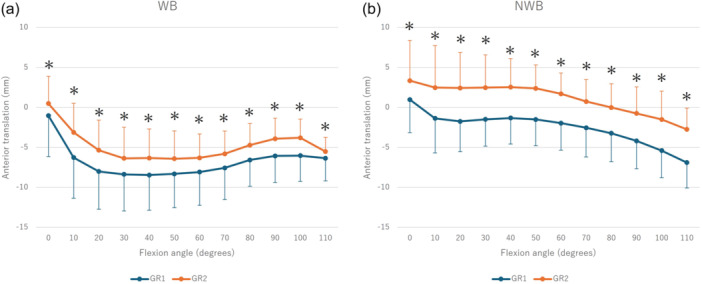
Kinematic comparison between Groups 1 and 2 regarding lateral anteroposterior translation during WB (a) and NWB (b). *Significant difference between Groups 1 and 2 (*p* < 0.05). NWB, non‐weight‐bearing; WB, weight‐bearing.

In the kinematic analysis, the knee externally rotated from 0° to 70° and from 50° to 110° of flexion during WB and NWB, respectively, with GR1 exhibiting greater external rotation across all ranges of motion (WB: *p* < 0.01, NWB: *p* < 0.01) (Figure 1). Medial contact points moved backwards from 0° to 30° of flexion during WB, forward from 30° to 100° of flexion, and backwards from 100° to 110° of flexion. Although GR1 was more forward throughout the full range of motion, no significant movement was observed during NWB (Figure 2). The lateral contact points moved backwards at 0−30° of flexion, forward at 70−100° of flexion and backwards at 100−110° of flexion during WB, while there was backward movement at 50° of flexion during NWB. Both activities were more backward in GR1 throughout the full range of motion (Figure 3).

## DISCUSSION

The most important finding of this study was that the FC in GR1 was externally rotated across all ranges of motion, and the lateral contact points were posterior in BCR‐TKA. Although there was no significant difference, FC tended to be internally rotated in GR2 in the rotational alignment between components at 2 weeks post‐operatively. The average duration of fluoroscopic surveillance was 11.2 ± 5.0 months in GR1 and 9.5 ± 2.1 months in GR2. Several longitudinal studies have reported that the femoral axial rotation after TKA tends to rotate externally over time [19, 24]. The femur is more externally rotated than normal knees at extension after BCR‐TKA [14]. Therefore, better clinical outcomes may be achieved by having the TC more externally rotated or the FC more internally rotated within the limits of coverage, aiming for a placement that facilitates screw‐home movement while minimising excessive external rotation and lateral posterior movement of the FC. A previous study reported that patients with higher PROM scores after BCS‐TKA experience less external rotation of the femur in deep knee flexion [13]. In addition, personalised BCR‐TKA, which adjusts for lateral joint laxity, provides superior short‐term clinical outcomes to unicompartmental knee arthroplasty [5]. BCR‐TKA also easily influences the lateral collateral ligament force [8]. Collectively, these findings suggest that proper lateral ligament balance is important in BCR‐TKA.

In this study, there were no significant differences between the two groups in anterior stability at 30° of flexion using the KT2000 arthrometer and medial contact points during the NWB activity. In contrast, the medial contact points during WB in GR2 were positioned more posteriorly. The anterior stability of the BCS‐TKA is positively linked with intraoperative medial stability [4]. Moreover, a previous study reported that patients with poor PROM scores after TKA experience more anterior translation on the medial side during WB [25]. In contrast, Mizu‐Uchi et al. reported that post‐operative proper valgus laxity and medial pivot kinematics are significantly associated with better clinical outcomes [16]. These findings suggest that the medial compartment also requires moderate laxity under specific WB conditions.

Similar to the kinematics observed in the present study, a previous study reported that the AP position of the medial and lateral contact points of the BCR‐TKA is significantly more posterior in the mid‐range of knee flexion in WB than in NWB, although no anterior translation was observed [12]. Nabeki et al. reported that the in situ force of the ACL and posterior cruciate ligament (PCL) increased after BCR‐TKA in vitro [17]. In contrast, in an in vivo study, following BCR‐TKA, only the PCL force was higher than that of normal knees and preoperative OA knees [14]. Furthermore, the lateral AP translation is positively correlated with the PCL force [9], suggesting that the excessive posterior location of lateral contact points in GR1 might contribute to increased PCL tightness, which is associated with lower PROMs. In the present study, the flexion angle in GR1 tended to be smaller than that in GR2. Perreault et al. reported that BCR‐TKA knees tend to exhibit reduced knee flexion [20]. Therefore, this PCL tightness may lead to reduced knee flexion.

This study had a few limitations. First, post‐operative kinematic data were collected only for one movement repetition per patient owing to ethical considerations aimed at minimising patients' x‐ray exposure as much as possible. However, the patients were asked to perform the task several times before data acquisition. Second, we analysed only high knee flexion activities. The other activities of daily living, such as gait or stair activities, might have displayed different kinematic changes. Finally, in the current study, an assessment for statistical correlations between rotational alignment and PROM scores was not performed. Previous studies demonstrated that rotational mismatch reduced clinical outcomes [7, 15].

In conclusion, the present study demonstrated that the FC in the low‐PROMs group was externally rotated across all ranges of motion, and the lateral contact points were posteriorly located in BCR‐TKA. Clinical outcomes may improve if the rotational alignment remains neutral, aiming to suppress excessive external rotation and lateral posterior movement of the FC.

## AUTHOR CONTRIBUTIONS

Kenichi Kono performed the study, carried out the formal analysis and wrote the manuscript. Hiroshi Inui carried out data curation and conceived the study. Takaharu Yamazaki, Ryota Yamagami and Tomofumi Kage provided technical assistance. Tetsuya Tomita, Shuji Taketomi and Sakae Tanaka provided general support. All authors read and approved the final manuscript.

## CONFLICT OF INTEREST STATEMENT

The authors declare no conflicts of interest.

## ETHICS STATEMENT

This study was performed in accordance with the principles of the Declaration of Helsinki. Approval was granted by the University of Tokyo Institutional Ethics Review Board (number 10462‐(1)).

## CONSENT

Informed consent was obtained from all participants included in the study. The patients also signed informed consent forms for the publication of their data and photographs.

## Data Availability

The data that support the findings of the current study are available from the corresponding author (Kenichi Kono) upon reasonable request. The data are not publicly available.
